# Acquired HIV-1 Drug Resistance and Molecular Transmission Networks in Zhongwei, Ningxia, China

**DOI:** 10.3390/v18060685

**Published:** 2026-06-18

**Authors:** Youping Duan, Subinuer Mutalifu, Ziyang Luo, Yufeng Li, Xiaohong Zhu, Jianxin Pei, Dongzhi Yang, Zhonglan Wu

**Affiliations:** 1 College of Public Health, Ningxia Medical University, Yinchuan 750004, China; 18609587418@163.com (Y.D.); 18295603903@163.com (S.M.); luoziyang99999@163.com (Z.L.); liyf0909@163.com (Y.L.); zxh_00921@163.com (X.Z.); 2 Ningxia Center for Disease Control and Prevention, Yinchuan 750011, China; peijianxin282@163.com (J.P.); nxcdcydz@126.com (D.Y.); 3 College of Life Sciences, University of Ningxia, Yinchuan 750021, China

**Keywords:** HIV-1, gene subtype, drug resistance, molecular transmission network

## Abstract

Objective: This retrospective cross-sectional study aimed to characterize HIV-1 genotypes, assess drug resistance, and analyze molecular transmission networks in Zhongwei City to inform prevention strategies. Methods: Plasma samples were collected from antiretroviral therapy (ART)-treated patients (2007–2024) with viral load ≥ 200 copies/mL. HIV-1 *pol* was amplified by nested PCR; successful sequences were genotyped by maximum likelihood (ML) (IQ-TREE, TVM+F+I+G4, 1000 bootstrap). Drug resistance (DR) was interpreted using Stanford HIV Drug Resistance Database (HIVDB) v9.0; detected mutations represent acquired drug resistance (ADR). Pairwise genetic distances (GD) (TN93 model) were calculated; transmission networks were constructed in Cytoscape 3.10.3. Results: 75 sequences were obtained. Males (84.00%), and heterosexual transmission (64.00%) predominated. CRF07_BC (46.67%) and CRF01_AE (38.67%) were the major subtypes; the overall ADR rate was 40.00%, mainly NNRTIs-associated (30.67% of all participants, including 16.00% single-class NNRTIs and 14.67% dual-class NRTIs-NNRTIs). Network inclusion rate was 40.00% of the 75 sequences; CRF07_BC showed higher betweenness centrality (*p* = 0.028), while CRF01_AE and CRF85_BC showed higher closeness centrality (*p* < 0.001). Occupation significantly affected network enrollment (*p* ≤ 0.05). Conclusion: HIV-1 subtypes are diverse with high ADR. CRF07_BC may act as a transmission bridge, whereas CRF01_AE and CRF85_BC exhibit faster potential spread. Baseline DR testing and network-guided interventions are recommended.

## 1. Introduction

Acquired Immunodeficiency Syndrome (AIDS) is a chronic viral illness resulting from infection with Human Immunodeficiency Virus (HIV). According to the Joint United Nations Program on HIV/AIDS (UNAIDS) statistics, by the end of 2024, 40.8 million people (37–45.6 million) worldwide were living with HIV, 31.6 million (27.8–32.9 million) were receiving antiretroviral therapy (ART), and 630,000 (490,000–820,000) died from AIDS-related illnesses [1]. These compelling epidemiological data underscore that HIV/AIDS remains a major public health issue requiring urgent global attention. As ART becomes increasingly widespread and coverage continues to expand both globally and in China, the issue of drug resistance has become increasingly prominent. This not only undermines the therapeutic efficacy of ART and increases the difficulty and cost of patient management but has also emerged as a key bottleneck that limits treatment outcomes and exacerbates the complexity of HIV prevention and control. It represents a significant challenge for both clinical practice and public health prevention and control efforts. Against this backdrop, China has been gradually refining its policies on drug resistance testing and treatment, providing clear guidance for local HIV drug resistance research and prevention and control efforts.

In 2016, China issued the “Notice on Adjusting the Standards for Free Antiviral Treatment for AIDS,” marking the nationwide implementation of the “treatment upon diagnosis” strategy [2]. The implementation of this strategy has significantly extended the life expectancy of HIV/AIDS patients. The World Health Organization (WHO) classifies HIV drug resistance into acquired drug resistance (ADR), transmitted drug resistance (TDR), and pretreatment drug resistance (PDR) based on when the resistance developed and was detected. ADR refers to drug-resistant mutations that arise during viral replication under drug pressure, TDR refers to drug resistance acquired through transmission in newly infected individuals, while PDR refers to drug resistance detected before initial or retreatment, which may originate from transmitted drug-resistant strains or prior exposure (such as mother-to-child transmission prevention, pre- and post-exposure prophylaxis, or ART interruption) [3]. According to the Chinese Guidelines for the Diagnosis and Treatment of AIDS (2024 Edition) [4] and the Chinese Expert Consensus on the Management of Antiviral Treatment Resistance in HIV Infection (2025) [5], standard baseline genotypic resistance testing should focus on the reverse transcriptase (RT) and protease (PR) regions of the HIV-1 *pol* gene. The RT region primarily reflects resistance to non-nucleoside reverse transcriptase inhibitors (NNRTIs) and nucleoside reverse transcriptase inhibitors (NRTIs), while the PR region corresponds to resistance to protease inhibitors (PIs). Testing of the integrase region is recommended only in “regions with limited resources” and for individuals with a history of exposure to integrase strand transfer inhibitors (INSTIs). In terms of treatment, the national free ART program continues to center on NNRTIs. According to the National Manual on Free Antiviral Treatment for HIV/AIDS (5th Edition, 2023) [6], the first-line regimen of choice is tenofovir disoproxil fumarate (TDF) + lamivudine (3TC) + efavirenz (EFV 400 mg). The alternative first-line regimen is TDF + 3TC + rilpivirine (RPV). The integrase inhibitor dolutegravir (DTG) is restricted to second-line treatment and requires resistance testing before use. A 2024 response letter from the National Center for Disease Control and Prevention also confirmed that DTG is a second-line drug. This means that as of the end of 2024, China’s national free first-line antiviral treatment remains NNRTIs–based, with INSTIs strictly restricted to second-line use. Of note, early regimens (before 2013) included stavudine (d4T) and didanosine (DDI), which were subsequently phased out. Against this backdrop, accurately identifying transmission hotspots and drug-resistant transmission chains has become key to prevention and control efforts. The emergence of molecular epidemiology has facilitated the extensive use of sequence–based molecular transmission networks for studying HIV-1. These networks offer new perspectives for exploring the transmission dynamics of HIV-1 and developing targeted intervention strategies for high-risk populations. Specifically, HIV-1 molecular transmission networks represent a novel approach to analyzing connection patterns, clustering mechanisms, and internal associations within transmission networks across specific populations [7,8,9].

Zhongwei City, a prefecture-level city under the jurisdiction of Ningxia Hui Autonomous Region, China, serves as a regional hub at the intersection of Ningxia, Gansu, and Inner Mongolia. As a major tourist destination, it is characterized by significant large-scale and frequent population movements. Against this national backdrop, this study focuses on the analysis of acquired drug resistance to three classes of antiretroviral drugs—NRTIs, NNRTIs, and PIs. By integrating molecular transmission networks, the study systematically analyzes the epidemiological characteristics, subtype distribution, and resistance levels of HIV-1 in Zhongwei City, with the aim of providing a scientific basis for local prevention and control strategies.

## 2. Materials and Methods

### 2.1. Sample Source

This was a retrospective cross-sectional study. We collected plasma samples from HIV/AIDS patients receiving ART in Zhongwei City between the first confirmed case in 2007 and the end of 2024. All plasma samples were stored at −80 °C. Viral load testing was performed by the laboratory at the Ningxia AIDS Confirmation Centre Laboratory.

Definition of virological non-suppression and sequencing threshold: According to domestic and international HIV treatment guidelines, a viral load of ≥ 200 copies/mL was used both as the criterion for virological non-suppression and as the laboratory threshold for HIV genotyping [10,11]. Based on multiple viral load follow-up records from the local AIDS Integrated Prevention and Control Information System, patients with a single detectable viral load that subsequently became <200 copies/mL (transient blip) were excluded [12]. Only patients with persistent viremia (viral load ≥ 200 copies/mL on consecutive follow-up measurements) were retained for sequencing and analysis [10].

Type of drug resistance mutation: Since all enrolled patients were on ART at the time of sampling and had viral loads ≥ 200 copies/mL (i.e., virologically non–suppressed), the resistance mutations detected in this study represent ADR, i.e., mutations emerging under selective drug pressure, rather than PDR.

**Figure 1:**
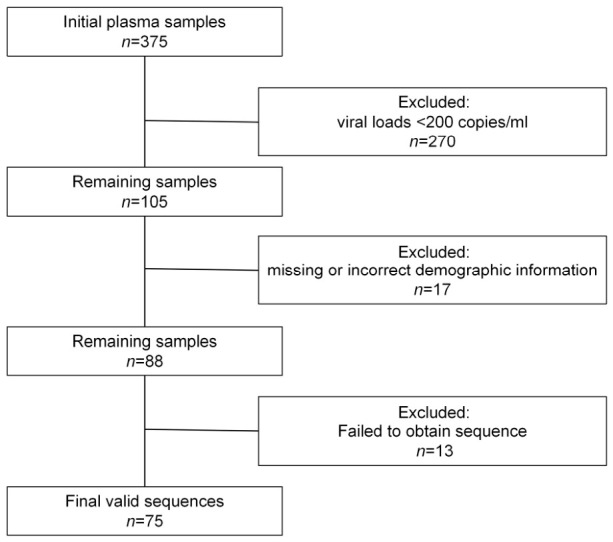
Sample inclusion and exclusion flowchart of Zhongwei City.

Sample selection and sequencing results: A total of 375 plasma samples were collected. A stepwise selection process was applied according to predefined criteria: (1) 270 cases with viral load < 200 copies/mL were excluded; (2) 17 cases with missing or incorrect demographic information were excluded. Finally, 88 samples with viral load ≥ 200 copies/mL and complete clinical data were selected for HIV-1 *pol* region sequencing. Among the 88 samples, 13 failed, and 75 yielded valid sequences (sequencing success rate 85.2%) (see Tables S1 and S2). Duplicated sequences from the same patient were excluded. The sample selection process is illustrated in Figure 1. For the 75 successfully sequenced cases, demographic and epidemiological data were extracted from the Ningxia AIDS Integrated Prevention and Control Information System for subsequent analysis.

### 2.2. Research Methods

#### 2.2.1. Nucleic Acid Extraction

Viral RNA was extracted from 200 μL of plasma using a fully automated nucleic acid extraction system (GeneRote X96, Tianlong Technology, Xi’an, China) and the corresponding kit (HIV-1 Nucleic Acid Extraction and Purification Kit, Xi’an Tianlong Technology Co., Ltd., Xi’an, China). An automated extraction program was set up in accordance with the instrument’s operating manual; the program included automatic lysis, washing, and elution, resulting in a final volume of 80 μL.

#### 2.2.2. Amplification of the HIV-1 *pol* Gene Region

The HIV-1 *pol* region (protease and reverse transcriptase, HXB2 2147–3462, ~1300 bp) was amplified by nested PCR. First-round RT–PCR (TransScript II One-Step Kit, TransGen, Beijing, China) used outer primers MAW26 (5′–TTGGAAATGTGGAAAGGAAGGAC–3′) and RT21 (5′–CTGTATTTCTGCTATTAAGTCTTTTGATGGG–3′). The 25 μL reaction mixture contained: 10.0 μL of R–MIX, 0.5 μL of E–MIX, 0.5 μL of MAW26, 0.5 μL of RT21, 8.5 μL of RNase-free water, and 5.0 μL of RNA template. Thermal cycling conditions were: 50 °C for 30 min, 94 °C for 5 min, 55 °C for 1 min, 72 °C for 2 min; then 30 cycles of 94 °C for 30 s, 50 °C for 45 s, 72 °C for 1 min 30 s; followed by a final extension at 72 °C for 10 min. Second–round nested PCR (2xTrans Taq HiFi SuperMix (TransGen, Beijing, China)) used inner primers PRO1 (5′–CAGAGCCAACAGCCCCACCA–3′) and RT20 (5′–CTGCCAGTTCTAGCTCTGCTTC–3′). The 50 μL reaction mixture contained: 25.0 μL of Mix, 1.0 μL of PRO1, 1.0 μL of RT20, 5.0 μL of the first-round PCR product, and 18.0 μL of nuclease–free water. Thermal cycling conditions were: 94 °C for 2 min; then 30 cycles of 94 °C for 30 s, 58 °C for 45 s, 72 °C for 1 min 30 s; followed by a final extension at 72 °C for 10 min. Amplified products were resolved on a 1% agarose gel, and positive amplicons (~1300 bp) were sent to Beijing Dehong Changyuan Co., Ltd. (Beijing, China) for purification and Sanger sequencing in both directions using the nested primers.

#### 2.2.3. Sequence Processing and Quality Control

Sequences containing ambiguous bases (>5% mixed signals) or with a quality score (Q) < 20 were excluded. The forward and reverse sequences were assembled and aligned using MEGA12 (version 12.0.11) software, with Clustal W for alignment and manual adjustments. The final aligned sequence was trimmed to remove gaps and uncertain regions at both ends.

#### 2.2.4. Phylogenetics and Genotyping

Phylogenetic trees were constructed using the maximum likelihood (ML) method with IQ-TREE 2.3.6 software. ModelFinder automatically selected the optimal nucleotide substitution model (TVM+F+I+G4, chosen based on the BIC criterion), and the analysis was bootstrapped 1000 times. Reference sequences were downloaded from the LANL HIV database (www.hiv.lanl.gov (accessed on 15 June 2026)), and subtypes were determined based on their clustering relationships with reference strains. Sequences that could not be clearly typed were further analyzed for recombination using RIP, jpHMM, or SimPlot.

#### 2.2.5. Resistance Analysis

Successfully sequenced HIV-1 *pol* sequences were submitted to the Stanford University HIV Drug Resistance Database (https://hivdb.stanford.edu/ (accessed on 15 June 2026), version HIVDB 9.0). Genotypic resistance was determined if low-level to higher resistance was detected for any drug.

#### 2.2.6. Construction of Molecular Transmission Networks

Using the MEGA12 (version 12.0.11) software, gene distances between all pairs of sequences were calculated based on the TN93 model. Molecular transmission networks were constructed within the range of 0.001 to 0.021 with an increment of 0.002. The minimum gene distance (0.011) that yielded the highest number of transmission clusters and a stable clustering structure was selected as the optimal threshold. Cytoscape 3.10.3 was used for network visualization.

### 2.3. Statistical Analysis

Data were entered into Excel and analyzed using SPSS 27.0. Comparisons between groups for categorical variables were performed using the chi-square test or Fisher’s exact test. Comparisons of degree centrality, betweenness centrality, and closeness centrality among different subtypes (CRF07_BC, CRF01_AE, CRF85_BC) in the molecular transmission network were performed using the Kruskal-Wallis H test. A two-sided *p* value < 0.05 was considered statistically significant.

## 3. Results

### 3.1. Demographic Characteristics

A total of 75 valid sequences were included in the subsequent analysis (see Figure 1 for the sample selection process). The viral loads of all successfully sequenced samples were ≥1000 copies/mL, indicating that samples with higher viral loads had a higher success rate. The main characteristics of this population are the following: male (63/75, 84.00%), residents of Shapotou District (40/75, 53.33%), aged 26–49 (35/75, 46.67%), married/partnered (38/75, 50.67%), junior high school education (25/75, 33.33%), and peasant (39/75, 52.00%). The primary mode of transmission was heterosexual transmission (48/75, 64.00%). Geographically, HIV/AIDS cases in Zhongwei City were mainly concentrated in Shapotou District. There was a statistically significant association between occupation and network enrollment (*p* = 0.019) (see Table 1).

### 3.2. Genotype Analysis

**Figure 2:**
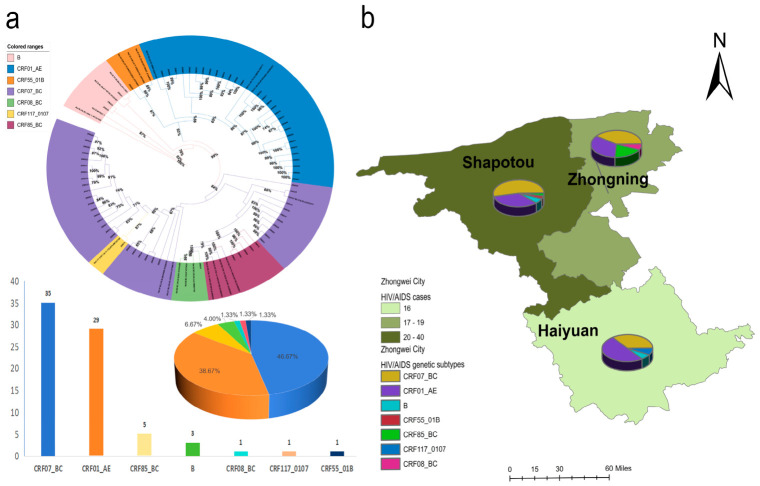
(**a**) Analysis of HIV-1 subtypes among HIV/AIDS patients in Zhongwei City; (**b**) distribution of HIV-1 Subtypes in Shapotou District, Zhongning County, and Haiyuan County, Zhongwei City, Ningxia.

A total of 7 genotypes were detected, including CRF07_BC (35/75, 46.67%), CRF01_AE (29/75, 38.67%), CRF85_BC (5/75, 6.67%), B (3/75, 4.00%), CRF117_0107 (1/75, 1.33%), CRF08_BC (1/75, 1.33%), and CRF55_01B (1/75, 1.33%). The phylogenetic tree is shown in Figure 2. The HIV-1 genotypic analysis results of this study indicate that CRF07_BC and CRF01_AE are predominant. Additionally, prevalent recombinant strains such as CRF55_01B and CRF117_0107 were detected.

Further analysis of the distribution of HIV-1 subtypes in Shapotou District, Zhongning County, and Haiyuan County showed that Shapotou District (40 cases) had five subtypes (CRF07_BC, CRF01_AE, B, CRF55_01B, CRF85_BC); Zhongning County (19 cases) had four subtypes (CRF07_BC, CRF01_AE, CRF85_BC, CRF08_BC); and Haiyuan County (16 cases) had four subtypes (CRF07_BC, CRF01_AE, B, CRF117_0107). Fisher’s exact test showed no significant difference in HIV-1 subtype distribution among the three regions (*χ*^2^ = 14.808, *p* = 0.126) (see Figure 2 and Table 2).

### 3.3. Drug Resistance Analysis

#### 3.3.1. Overall Resistance Analysis

**Figure 3:**
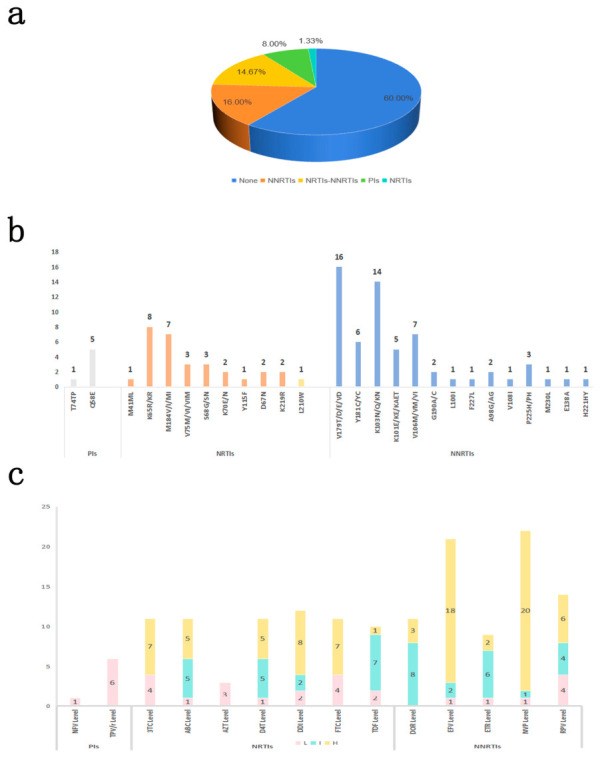
(**a**) Distribution and proportion of drug resistance types in the study population; (**b**) drug resistance mutations in HIV/AIDS patients; (**c**) antiretroviral drug resistance profile by level (L/I/H). Abbreviations: L, low-level resistance; I, intermediate-level resistance; H, high-level resistance.

This study included early-generation drugs (e.g., D4T, DDI, IDV/r) because the investigation started in 2007, when these agents had not yet been phased out in China. Their inclusion therefore reflects the actual treatment landscape during the study period. Among the 75 patients with successfully obtained genetic sequences, 30 were identified as having ADR, giving an overall ADR rate of 40.00% (30/75). The distribution of resistance types was as follows: single-class NNRTIs resistance in 12 cases (16.00%), single-class NRTIs resistance in 1 case (1.33%), single-class PIs resistance in 6 cases (8.00%), and dual-class (NRTIs-NNRTIs) resistance in 11 cases (14.67%) (see Figure 3).

Among the 30 resistant patients, the predominant PIs mutation was Q58E with 5 cases, and all PIs drugs (NFV, TPV/r) showed only low-level resistance. Total NRTIs mutation counts included NRTIs mutations from both the single-class NRTIs–resistant group and the dual-class resistant group, with the major mutations being K65R/KR with 8 cases and M184V/I/MI with 7 cases; high-level NRTIs resistance was concentrated on DDI, 3TC, and FTC, while intermediate-level resistance was mainly observed with TDF, ABC, and D4T. Total NNRTIs mutation counts included NNRTIs mutations from both the single-class NNRTIs–resistant group and the dual-class resistant group, with the predominant mutations being V179T/D/E/VD with 16 cases, K103N/Q/KN with 14 cases, V106M/VM/VI with 7 cases, Y181C/YC with 6 cases, and K101E/KE/KAET with 5 cases. High-level NNRTIs resistance was most prominent for NVP with 20 cases and EFV with 18 cases (see Figure 3).

#### 3.3.2. Analysis of Resistance Characteristics by Resistance Type

**Figure 4:**
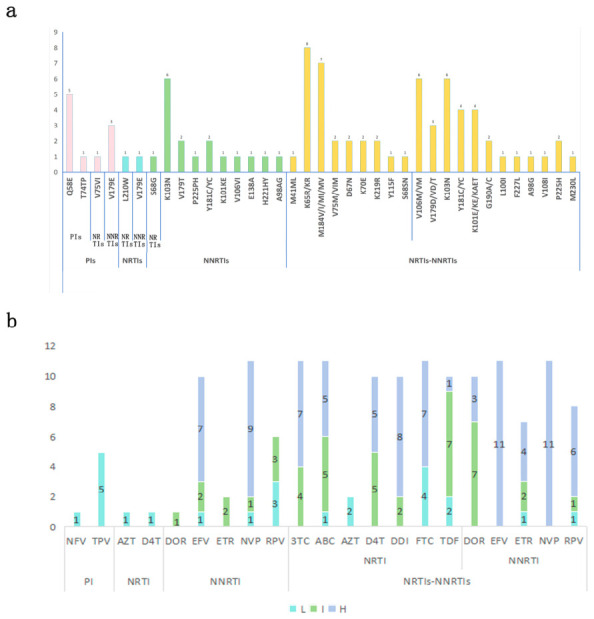
(**a**) Drug resistance mutations by resistance type and their frequency; (**b**) antiretroviral drug use by resistance type (stratified by resistance level L/I/H).

The single-class PIs-resistant group, consisting of 6 cases, had the predominant mutation Q58E in 5 cases (83.33%), with low-level resistance to NFV and TPV. The single-class NRTIs–resistant group, 1 case, carried L210W and V179E (50.00%) and showed low-level resistance only to AZT and D4T. Among the single-class NNRTIs–resistant group of 12 cases, the main presented mutations were K103N in 6 cases (50.00%), V179T in 2 cases (16.67%), and Y181C in 2 cases (16.67%); high-level resistance was observed for EFV in 7 cases (58.33%) and NVP in 9 cases (75.00%). The dual-class resistant group of 11 cases included NRTIs mutations K65R/KR in 8 cases (72.73%) and M184V/I/MI/MV in 7 cases (63.64%), and NNRTIs mutations K103N and V106M/VM in 6 cases each (54.55%); all 11 cases (100.00%) showed high-level resistance to both EFV and NVP, and high-level resistance to NRTIs drugs was frequent for DDI in 8 cases (72.73%), and for 3TC and FTC in 7 cases each (63.64%) (see Figure 4).

### 3.4. Molecular Transmission Network Analysis

**Figure 5:**
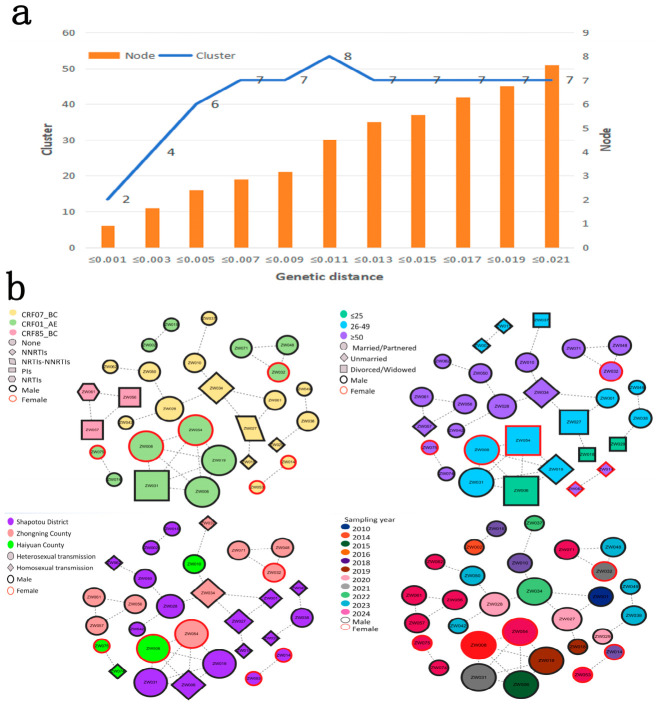
(**a**) The number of nodes and clusters at different genetic distances; (**b**) analysis of the genetic transmission network among HIV/AIDS patients in Zhongwei City.

After screening values ranging from 0.001 to 0.021 (in 0.002 increments), the optimal genetic distance threshold was determined to be 0.011. Below this threshold, the constructed molecular transmission network comprises 30 nodes (representing 40.00% of the 75 sequences) and 31 edges, forming 8 transmission clusters (Figure 5b). In the molecular transmission network, participants were predominantly male (24/30, 80.00%), heterosexual transmission was the most common route of infection (20/30, 66.67%), and the age group of 50 years and older accounted for the highest proportion (16/30, 53.33%). A total of three HIV-1 subtypes were detected within the network: CRF07_BC (15/30, 50.00%), CRF01_AE (12/30, 40.00%), and CRF85_BC (3/30, 10.00%). Drug resistance analysis revealed that drug resistance was detected in 8 sequences within the network (8/30, 26.67%); resistance patterns included NNRTIs, NRTIs-NNRTIs, PIs, and NRTIs. When stratified by sampling year, 70.0% (21/30) of networked nodes were from 2019–2024, and only 10.0% (3/30) were from 2007–2015, indicating that recent cases constitute the main component of the network. The remaining six nodes (20.0%) were sampled between 2016 and 2018.

This study identified two major transmission clusters. One is the large CRF07_BC cluster centered on ZW034, comprising 10 male cases, predominantly aged ≥50 years (6/10, 60.00%), distributed across Shapotou District, Zhongning County, and Haiyuan County. Transmission routes included both heterosexual transmission and homosexual transmission, with drug resistance detected in 3 cases (NNRTIs and NRTIs-NNRTIs). The nodes span the years 2010, 2014, 2018, 2020, 2022, 2023, and 2024, forming a continuous transmission chain spanning at least 14 years. The other is the CRF01_AE core cluster, comprising five cases (ZW008, ZW054, ZW019, ZW006, and ZW031), consisting of 2 females and 3 males, predominantly aged 26–49 years (4/5, 80.00%), this case also spans three districts and counties, involves one case carrying PIs resistance, and covers the period from 2015 to 2024, indicating sustained transmission over multiple years.

Centrality analysis (Table 3) showed that the mean degree centrality for the three subtypes (CRF07_BC, CRF01_AE, and CRF85_BC) was 1.73 ± 0.96, 2.50 ± 1.38, and 2.00 ± 0.00, respectively. The Kruskal–Wallis test revealed no statistically significant differences among the three groups (H = 2.509, *p* = 0.285). The mean betweenness centrality values were 0.20 ± 0.32, 0.00 ± 0.00, and 0.00 ± 0.00, respectively, with statistically significant differences between groups (H = 7.147, *p* = 0.028). The mean values for closeness centrality were 0.56 ± 0.26, 1.00 ± 0.00, and 1.00 ± 0.00, respectively, with highly significant differences between groups (H = 17.775, *p* < 0.001).

## 4. Discussion

This study is a retrospective cross-sectional study of HIV-1/AIDS patients in Zhongwei City. It represents the first systematic epidemiological and molecular characterization of all reported cases from 2007 to 2024. The study included 75 individuals from whom HIV-1 *pol* region sequences were successfully obtained. Their demographic characteristics are as follows: men (84.00%), and the primary transmissions of heterosexual (64.00%). This finding aligns with previous reports from this geographic area [13,14]. Studies in other regions of China have also identified this trend of a higher proportion of males among HIV-1/AIDS cases, with heterosexual transmission being one of the primary modes of transmission of infection [15,16].

In terms of genetic subtypes, this study identified a total of seven HIV-1 genotypes (CRF07_BC, CRF01_AE, B, CRF08_BC, CRF85_BC, CRF55_01B, CRF117_0107). CRF07_BC and CRF01_AE emerged as the predominant subtypes, aligning with findings reported from other regions across China [17,18,19]. Since the first case of HIV infection was reported in Zhongwei City in 2007, the novel recombinant strains CRF55_01B and CRF117_0107 have been detected successively, suggesting that the introduction of foreign strains and the evolution of local strains are accelerating. Further analysis of subtype distribution across the three counties and districts revealed that Shapotou District showed a high concentration of CRF07_BC and CRF01_AE (combined 90.0%), Zhongning County exhibited four co–circulating subtypes (CRF07_BC, CRF01_AE, CRF85_BC, and CRF08_BC), while Haiyuan County was dominated by CRF01_AE (50.0%). The differences in distribution among the three areas were not statistically significant (*χ*^2^ = 14.808, *p* = 0.126). Nevertheless, the detection of multiple recombinant forms (CRF85_BC, CRF08_BC, CRF55_01B, CRF117_0107) indicates ongoing viral evolution and recombination activity in this region. Therefore, continuous molecular surveillance of HIV-1 subtypes and emerging recombinants is necessary to monitor transmission dynamics and inform prevention strategies.

This study is a retrospective cross-sectional analysis of ADR among HIV-1/AIDS patients receiving antiretroviral therapy in Zhongwei City. It should be noted that the study period (2007–2024) spans the evolution of China’s National List of Free Antiviral Medications. The test panel used in this study includes some early-generation drugs that have since been discontinued in China; these drugs were still part of the free treatment regimen at the beginning of the study, and their resistance data accurately reflect the treatment landscape at that time [20,21]. Furthermore, since routine drug resistance testing in China targets only the protease and reverse transcriptase regions, and integrase strand transfer inhibitors (INSTIs) were not widely available in Ningxia during the study period, this study did not assess INSTI resistance [4,6]. Therefore, the exclusion of INSTI resistance data does not affect the main conclusions of this study. HIV drug resistance mutations were detected in 30 individuals, and the drug resistance rate was 40.00% (30/75). This aligns with the drug resistance rate among HIV-1 infected individuals in Ningxia during 2020–2023 (221/544, 40.06%) [22] and the resistance rate among heterosexually transmitted cases in Ningxia during 2020–2021 (109/269, 40.52%) [13], but lower than that observed in certain provinces and municipalities within China with high resistance rates [23,24], indicating a regional difference in HIV-1 prevalence. The most prevalent resistance pattern observed in this study was monoresistance to NNRTIs, followed by dual resistance to both NNRTIs and NRTIs and monoresistance to PIs. Among the drug resistance mutations in NNRTIs, K103N were the main drug resistance sites. Studies have shown that mutations at the K103N resistance site can lead to high levels of resistance to NVP and EFV in patients receiving ART [25]. This means that individuals with such mutations face a higher risk of virological failure if treated with standard first-line regimens. To mitigate this risk, it is crucial to determine the patient’s resistance profile before treatment. At the same time, regardless of the presence of baseline resistance, good treatment adherence during therapy is the key to preventing the accumulation and transmission of new resistance mutations. Therefore, it is recommended that baseline resistance testing be fully implemented in this region, accompanied by strengthened regular follow-up and adherence support, to optimize individualized treatment strategies, reduce the rate of virological failure, and prevent the further spread of resistant strains. Although domestic and international guidelines recommend routine baseline drug resistance testing for treatment–naïve patients, its implementation in primary healthcare institutions in Zhongwei was previously limited due to insufficient laboratory capacity, funding, and staffing. In recent years, local health authorities have launched a specialized program for routine baseline HIV drug resistance surveillance, improved laboratory networks, and gradually implemented standardized pre-treatment genotypic resistance testing for newly diagnosed cases. Therefore, it is essential to continuously promote baseline resistance testing and strengthen laboratory capacity in this region.

In this study, a molecular transmission network was constructed using an optimal genetic distance of 0.011, with a network inclusion rate of 40.00% (30/75), resulting in the formation of eight transmission clusters. The two main transmission clusters exhibit distinct temporal distribution patterns: The CRF07_BC cluster spans from 2010 to 2024, lasting over 14 years. It primarily involves middle–aged and elderly men aged 50 and older and spans three districts and counties, suggesting that this subtype has established a relatively persistent local transmission chain among the elderly population. This characteristic aligns with the recent trend of an increasing proportion of HIV cases among the elderly in China [26,27]; In contrast, the CRF01_AE cluster is dominated by young and middle–aged adults aged 26–49, with its active period concentrated after 2015, and it similarly exhibits cross–year transmission characteristics. Centrality analysis revealed that CRF07_BC had the highest betweenness centrality, suggesting it may serve as a link between different transmission clusters, while CRF01_AE and CRF85_BC had higher closeness centrality, indicating they may possess greater transmission efficiency. In addition, some male nodes in this study who reported heterosexual transmission were directly linked to MSM nodes. This phenomenon may be attributed to two factors: first, some MSM conceal their true sexual orientation or behavior due to social stigma and self–report heterosexual transmission in epidemiological surveys; second, the presence of bisexual behavior, meaning these men engage in unprotected sex with both men and women, thereby serving as “bridge” individuals connecting the MSM population with the general heterosexual population [28,29,30,31]. Such “key individuals” possess high centrality within transmission networks; their presence may break down transmission barriers between different high-risk populations, accelerating the spread of HIV from MSM populations to the general population. Therefore, molecular network surveillance should focus on these “bridge” nodes with prominent network positions, conducting in-depth case interviews and behavioral surveys to clarify the actual transmission chains [32]. In terms of geographic distribution, the nodes of this transmission cluster are located in Shapotou District, Zhongning County, and Haiyuan County, indicating that HIV-1 has spread beyond the boundaries of a single county and is showing a trend of cross-regional transmission. Population mobility (such as for work, education, or tourism) and convenient transportation (including the Bao–Lan Railway and the Beijing–Lhasa Expressway) may be key factors driving the cross-regional spread of the virus. Of particular concern is that drug resistance mutations against NNRTIs or NRTIs-NNRTIs combinations have been detected in some nodes within this transmission cluster (e.g., ZW034, ZW027), suggesting that drug-resistant strains may be spreading across regions. This further underscores the necessity of conducting baseline drug resistance testing and ongoing viral load monitoring to enable timely adjustments to treatment regimens and curb the further spread of drug-resistant strains. In summary, the HIV-1 molecular transmission network in Zhongwei City exhibits a complex structure centered on the MSM population, radiating to heterosexual populations through bisexual individuals or MSM who have not disclosed their status. It is recommended that molecular network analysis be deeply integrated with epidemiological investigations to implement targeted interventions at key nodes within the network (such as enhanced testing, promotion of pre-exposure prophylaxis, and partner tracing), while simultaneously conducting joint prevention and control efforts for clusters with cross-regional transmission to halt the continued spread of HIV-1.

This study has several limitations. First, due to the retrospective cross-sectional design, the sample size is relatively small (75 sequences) and the study period is long (2007–2024), which precludes a reliable temporal trend analysis. Second, the sequencing success rate was 85.2% (75/88), and the failure of some samples may be related to viral load or sample storage conditions. Third, we sequenced only the PR and RT regions and did not analyse the integrase region; therefore, INSTI resistance could not be assessed. Fourth, due to the retrospective nature of the surveillance data, detailed individual ART histories could not be retrieved. Future prospective studies with whole-genome sequencing are needed to further validate and extend our findings.

## 5. Conclusions

The HIV-1 epidemic in Zhongwei City is dominated by CRF07_BC and CRF01_AE, with multiple recombinant strains co-circulating. The ADR rate is 40.00%, with NNRTI resistance being the most prevalent. The molecular transmission network is active, exhibits cross-regional and cross-year transmission, and includes both heterosexual and homosexual transmission routes. Centrality analysis reveals that CRF07_BC may act as a transmission bridge, whereas CRF01_AE and CRF85_BC show higher potential for rapid spread. Baseline drug resistance testing and molecular network-guided interventions at key nodes are recommended.

## Figures and Tables

**Table 1:** Demographic Characteristics and Annual Distribution of HIV/AIDS Patients in Zhongwei City.

Characteristic	Total (*n* = 75)	Enter Network (*n* = 30)	Not Enter Network (*n* = 45)	*χ* ^2^	*p*
Gender (*n*%)				–	0.526
Female	12 (16.00)	6 (20.00)	6 (13.33)		
Male	63 (84.00)	24 (80.00)	39 (86.67)		
Age (Years) (*n*%)				5.393 *	0.075
≤25	12 (16.00)	3 (10.00)	9 (20.00)		
26–49	35 (46.67)	11 (36.67)	24 (53.33)		
≥50	28 (37.33)	16 (53.33)	12 (26.67)		
Place of residence				2.034	0.385
Shapotou District	40 (53.33)	17 (56.67)	23 (51.11)		
Zhongning County	19 (25.33)	9 (30.00)	10 (22.22)		
Haiyuan County	16 (21.33)	4 (13.33)	12 (26.67)		
Route of infection (*n*%)				0.154	0.808
Heterosexual transmission	48 (64.00)	20 (66.67)	28 (62.22)		
Homosexual transmission	27 (36.00)	10 (33.33)	17 (37.77)		
Marital status (*n*%)				2.118	0.347
Married/Partnered	38 (50.67)	17 (56.67)	21 (46.67)		
Unmarried	22 (29.33)	6 (20.00)	16 (35.55)		
Divorced/Widowed	15 (20.00)	7 (23.33)	8 (17.78)		
Educational level (*n*%)				5.266 *	0.263
Illiterate	8 (10.67)	4 (13.33)	4 (8.89)		
Primary school	18 (24.00)	7 (23.33)	11 (24.44)		
Junior high school	25 (33.33)	13 (43.33)	12 (26.67)		
High school/vocational school	11 (14.67)	4 (13.33)	7 (15.56)		
College degree or above	13 (17.33)	2 (6.67)	11 (24.44)		
Occupation (*n*%)				9.823 *	0.019
Peasant	39 (52.00)	18 (60.00)	21 (46.67)		
Household chores and unemployment	10 (13.33)	3 (10.00)	7 (15.56)		
Employee	10 (13.33)	7 (23.33)	3 (6.67)		
Others	16 (21.33)	2 (6.67)	14 (31.11)		
Subtype (*n*%)				0.848 *	0.728
CRF01_AE	29 (38.67)	12 (40.00)	17 (37.77)		
CRF07_BC	35 (46.67)	15 (50.00)	20 (44.44)		
Others	11 (14.67)	3 (10.00)	8 (17.78)		
Drug resistance (*n*%)				3.704	0.091
No	45 (60.00)	22 (73.33)	23 (51.11)		
Yes	30 (40.00)	8 (26.67)	22 (48.89)		

Note: * Fisher’s exact test (2 × 2) or Fisher–Freeman–Halton test (*r* × 2) was used; ‘–’ indicates *χ*^2^ not applicable.

**Table 2:** Distribution of HIV-1 subtypes among three districts of Zhongwei City.

Place of Residence	HIV-1 Subtypes							*χ* ^2^	*p*
	CRF07_BC	CRF01_AE	CRF85_BC	CRF117_0107	B	CRF08_BC	CRF55_01B		
Shapotou District	21	15	1	0	2	0	1	14.808 *	0.126
Zhongning County	8	6	4	0	0	1	0		
Haiyuan County	6	8	0	1	1	0	0		

Note: * If the expected value of the grid is less than 5, use Fisher’s test for statistical analysis.

**Table 3:** Centrality measures of HIV-1 subtypes in the molecular transmission network.

Parameter	CRF07_BC (*n* = 15)	CRF01_AE (*n* = 12)	CRF85_BC (*n* = 3)	Kruskal–Wallis H	*p* Value
Degree	1.73 ± 0.96	2.50 ± 1.38	2.00 ± 0.00	2.509	0.285
Betweenness	0.20 ± 0.32	0.00 ± 0.00	0.00 ± 0.00	7.147	0.028
Closeness	0.56 ± 0.26	1.00 ± 0.00	1.00 ± 0.00	17.775	<0.001

## Data Availability

The datasets used and/or analyzed during the current study are available from the corresponding author on reasonable request.

## Supplementary Materials

The following supporting information can be downloaded at: https://www.mdpi.com/article/10.3390/v18060685/s1, Table S1: Viral load values and failure reasons for samples with failed HIV-1 pol sequencing (viral load ≥ 200 copies/mL); Table S2: Sampling date, viral load, address, and HIV-1 subtype of HIV/AIDS patients in Zhongwei City.

## Abbreviations

The following abbreviations are used in this manuscript:

AIDS	Acquired Immunodeficiency Syndrome
HIV	Human Immunodeficiency Virus
UNAIDS	The Joint United Nations Program on HIV/AIDS
MSM	Men who have sex with men
ART	Antiretroviral therapy
PIs	Protease inhibitors
NRTIs	Nucleoside reverse transcriptase inhibitors
NNRTIs	Non-nucleoside reverse transcriptase inhibitors
INSTI	Integrase Strand Transfer Inhibitor
ATV	Atazanavir
DRV	Darunavir
FPV	Fosamprenavir
IDV	Indinavir
LPV	Lopinavir
NFV	Nelfinavir
SQV	Saquinavir
TPV	Tipranavir
3TC	Lamivudine
ABC	Abacavir
AZT	Zidovudine
D4T	Stavudine
DDI	Didanosine
FTC	Emtricitabine
TDF	Tenofovir disoproxil fumarate
DOR	Doravirine
EFV	Efavirenz
ETR	Etravirine
NVP	Nevirapine
RPV	Rilpivirine

## References

[1] Global HIV & AIDSStatistics—Fact Sheet. https://www.unaids.org/en/resources/fact-sheet.

[2] Poon A.F., Gustafson R., Daly P., Zerr L., Demlow S.E., Wong J., Woods C.K., Hogg R.S., Krajden M., Moore D. (2016). Near real-time monitoring of HIV transmission hotspots from routine HIV genotyping: An implementation case study. Lancet HIV.

[3] HIV Drug Resistance Report 2017. https://www.who.int/publications/i/item/9789241512831.

[4] Acquired Immunodeficiency Syndrome Professional Group, Society of Infectious Diseases, Chinese Medical Association, Chinese Center for Disease Control and Prevention (2024). Chinese Guidelines for the Diagnosis and Treatment of HIV/AIDS (2024 Edition). J. Peking. Union. Med. Coll..

[5] Zhang H.X., Chen L., Chen W.P., Chen W., Chen Y.K., Huang Y., Jiang J., Li H., Li L.H., Li X. (2025). Expert consensus on the management of antiretroviral drug resistance in HIV infection in China. Chin. J. AIDS STD.

[6] National Center for AIDS/STD Control and Prevention (NCAIDS), Chinese Center for Disease Control and Prevention (China CDC) (2023). National Handbook on Free Antiviral Treatment for HIV/AIDS.

[7] Ávila-Ríos S., García-Morales C., Valenzuela-Lara M., Chaillon A., Tapia-Trejo D., Pérez-García M., López-Sánchez D.M., Maza-Sánchez L., Del Arenal-Sánchez S.J., Paz-Juárez H.E. (2019). HIV-1 drug resistance before initiation or re-initiation of first-line ART in eight regions of Mexico: A sub-nationally representative survey. J. Antimicrob. Chemother..

[8] Ragonnet-Cronin M., Hu Y.W., Morris S.R., Sheng Z., Poortinga K., Wertheim J.O. (2019). HIV transmission networks among transgender women in Los Angeles County, CA, USA: A phylogenetic analysis of surveillance data. Lancet HIV.

[9] Wertheim J.O., Leigh Brown A.J., Hepler N.L., Mehta S.R., Richman D.D., Smith D.M., Kosakovsky Pond S.L. (2014). The global transmission network of HIV-1. J. Infect. Dis..

[10] Panel on Antiretroviral Guidelines for Adults and Adolescents, U.S. Department of Health and Human Services Guidelines for the Use of Antiretroviral Agents in Adults and Adolescents with HIV. https://clinicalinfo.hiv.gov/sites/default/files/guidelines/archive/adult-adolescent-arv-2024-09-12.pdf.

[11] Acquired Immunodeficiency Syndrome Professional Group, Society of Infectious Diseases, Chinese Medical Association, Chinese Center for Disease Control and Prevention (2024). Chinese guidelines for the diagnosis and treatment of human immunodeficiency virus infection/acquired immunodeficiency syndrome (2024 edition). Chin. Med. J. (Engl.).

[12] Grochowski J., Saberi P., Tseng A.L., Sherman E.M., Lam J.T. (2025). HIV Treatment Failure and Resistance. HIV Pharmacotherapy: Clinical Approaches and Applications.

[13] Pei J., Wu Z., Si B., Ma C., Liu Y., Ma X., Kuai W., Zhang Y., Li Y. (2024). HIV-1 drug resistance and genetic transmission networks among patients with sexually transmitted HIV in Ningxia, China. Front. Public Health.

[14] Wu Z., Liu Y., Ma X., Li Y., Zhu X., Yang D., Pei J., Li Y. (2025). Epidemiological and spatiotemporal analysis of elderly HIV-1/AIDS patients in Ningxia, China, from 2018 to 2023. Sci. Rep..

[15] Gao R., Li W., Xu J., Guo J., Wang R., Zhang S., Zheng X., Wang J. (2024). Characteristics of Subtype and Molecular Transmission Networks among Newly Diagnosed HIV-1 Infections in Patients Residing in Taiyuan City, Shanxi Province, China, from 2021 to 2023. Viruses.

[16] Huang T., He J., Su Q., Wei L., Qin J., Huang X., Tao C., Zhang F., Ye L., Cen P. (2025). Molecular epidemiology and pretreatment drug resistance of HIV-1 among newly diagnosed individuals in Nanning City, Guangxi, China. Microbiol. Spectr..

[17] Wu Z.L., Guan G.Y., Zhao J.H., Ma X.M., Wang X.M., Yang D.Z., Cao M., Rawle D.J. (2018). Dynamic Characteristics and HIV Infection of Men who have Sex with Men from 2011 to 2017 in Yinchuan, Ningxia, China. Curr. HIV Res..

[18] Zhou P.P., Yu G., Kuang Y.Q., Huang X.H., Li Y., Fu X., Lin P., Yan J., He X. (2019). Rapid and complicated HIV genotype expansion among high-risk groups in Guangdong Province, China. BMC Infect. Dis..

[19] Yu D., Li M., Wei L., Zhu K., Zhang R., Luo T., Ning Y., Liang H., Zhang J., Ye L. (2025). High genetic diversity of HIV-1 pol region and molecular transmission networks among people living with HIV-1 in Haikou, South China, 2005–2022. BMC Infect. Dis..

[20] Chuntao M., Qiang W., Yukun Z., Xianyu M. (2021). Analysis on the composition of free antiviral drugs for AIDS treatment inChina from 2009 to 2019. China Med. Her..

[21] Dou Z., Zhang F., Zhao Y., Jin C., Zhao D., Gan X., Ma Y. (2015). [Progress on China’ s national free antiretroviral therapy strategy in 2002–2014]. Zhonghua Liu Xing Bing Xue Za Zhi.

[22] Li Y. (2025). Analysis of Drug Resistance and Molecular Transmission Network of HIV-1/AIDS Patients in Ningxia. Master’s Thesis.

[23] Kong L.H., Xie X.X., Fu Y.H., Lin G., Yang X.Y., Ma S.J., Li H. (2025). Pre-treatment and Acquired Antiretroviral Drug Resistance amongPeople Living with HlV in Southwest China. Chin. Gen. Pract. Med..

[24] Xiao S., Liu T., Jiang Y.F., Li Y., He S., Yang L., Tan X., Xu C., Sun W. (2022). Analysis of viral genetic characteristics and drug resistance among 588newly reported HlV-1 patients in Heilongjiang Province from 2019 to2021. Chin. J. AIDS STD.

[25] Ávila-Ríos S., García-Morales C., Matías-Florentino M., Romero-Mora K.A., Tapia-Trejo D., Quiroz-Morales V.S., Reyes-Gopar H., Ji H., Sandstrom P., Casillas-Rodríguez J. (2016). Pretreatment HIV-drug resistance in Mexico and its impact on the effectiveness of first-line antiretroviral therapy: A nationally representative 2015 WHO survey. Lancet HIV.

[26] Guan Y., Zhu H., Qi T., Zhang R., Chen J., Liu L., Shen Y., Lu H., Tang Q. (2022). HIV/AIDS strategies should focus on outcomes and the psychological status of older patients diagnosed with HIV. Biosci. Trends.

[27] Hou Y., Jin Y., Cai C., Qin Q., Tang H., Lyu F. (2023). Comparative Analysis of Epidemiological Features of HIV/AIDS Cases Aged Over and Under 50 Years Old—China, 2010–2022. China CDC Wkly..

[28] Chen Y., Lan G., Feng Y., Ruan Y., Shen Z., McNeil E.B., Tang K., Huang J., Shao Y., Lin M. (2023). Inferring potential non-disclosed men who have sex with men among self-reported heterosexual men with HIV in Southwest China: A genetic network study. PLoS ONE.

[29] Tang W., Mao J., Tang S., Liu C., Mollan K., Cao B., Wong T., Zhang Y., Hudgens M., Qin Y. (2017). Disclosure of sexual orientation to health professionals in China: Results from an online cross-sectional study. J. Int. AIDS Soc..

[30] Yan H., He W., Huang L., Wu H., Liang Y., Li Q., Shui J., Wang C., Dzakah E.E., Han Z. (2020). The Central Role of Nondisclosed Men Who Have Sex With Men in Human Immunodeficiency Virus-1 Transmission Networks in Guangzhou, China. Open Forum Infect. Dis..

[31] Zhuoma L., Zhang Y., Yan T., Kang F., Hou X., Chen J., Huang M., Zeng Y., Wang Q., Zhou C. (2022). Non-disclosed men who have sex with men within local MSM HIV-1 genetic transmission networks in Guangyuan, China. Front. Public Health.

[32] Ragonnet-Cronin M., Hué S., Hodcroft E.B., Tostevin A., Dunn D., Fawcett T., Pozniak A., Brown A.E., Delpech V., Brown A.J.L. (2018). Non-disclosed men who have sex with men in UK HIV transmission networks: Phylogenetic analysis of surveillance data. Lancet HIV.

