# Supplementary material for: Acquired HIV-1 Drug Resistance and Molecular Transmission Networks in Zhongwei, Ningxia, China
Source: Viruses. 2026 Jun 18;18(6):685. doi: 10.3390/v18060685 (PMC13307735; doi:10.3390/v18060685)
Supplement: Supplementary file 1 [file viruses-18-00685-s001.zip › Table S1.pdf]

Table S1. Viral load values and failure reasons for samples with failed HIV-1 *pol* sequencing (viral load ≥200 copies/mL).

Sample ID	Viral load (copies/ml)	Reasons for Failure
ZW076	4,000	PCR amplification failed
ZW077	960	PCR amplification failed
ZW078	411	No band on agarose gel
ZW079	230	Low viral load, PCR amplification failed
ZW080	2,920	PCR amplification failed
ZW081	390	Low viral load, PCR amplification failed
ZW082	19,532	PCR amplification failed
ZW083	38,300	PCR amplification failed
ZW084	18,500	Weak band and low sequencing signal
ZW085	4,003	No band on agarose gel
ZW086	1,290	Weak band and low sequencing signal
ZW087	29,580	No band on agarose gel
ZW088	61,845	PCR amplification failed
