# Supplementary material for: Acquired HIV-1 Drug Resistance and Molecular Transmission Networks in Zhongwei, Ningxia, China
Source: Viruses. 2026 Jun 18;18(6):685. doi: 10.3390/v18060685 (PMC13307735; doi:10.3390/v18060685)
Supplement: Supplementary file 1 [file viruses-18-00685-s001.zip › Table S2.pdf]

Table S2. Sampling date, viral load, address, and HIV-1 subtype of HIV/AIDS patients in Zhongwei City.

Number	Sampling Date (Year)	Viral load (copies/ml)
ZW001	2010	90937
ZW002	2014	30434
ZW003	2015	19000
ZW004	2015	47300
ZW005	2015	70100
ZW006	2015	1935
ZW007	2015	45448
ZW008	2016	1030
ZW009	2016	7725
ZW010	2018	1117
ZW011	2018	1296
ZW012	2018	8224
ZW013	2018	48536
ZW014	2018	23548
ZW015	2018	28000
ZW016	2018	21405
ZW017	2019	1650
ZW018	2019	19242
ZW019	2019	4744
ZW020	2019	135671
ZW021	2019	1109904
ZW022	2019	76408
ZW023	2019	620733
ZW024	2019	2146791
ZW025	2020	33000
ZW026	2020	13961
ZW027	2020	2920
ZW028	2020	251165
ZW029	2020	4241
ZW030	2021	114101
ZW031	2021	340588
ZW032	2021	15408
ZW033	2021	38895
ZW034	2022	28000
ZW035	2022	12240
ZW036	2022	214483
ZW037	2022	1653
ZW038	2023	17669
ZW039	2023	168500
ZW040	2023	2677
ZW041	2023	130553

Number	Sampling Date (Year)	Viral load (copies/ml)
ZW042	2023	8573
ZW043	2023	176486
ZW044	2023	4004
ZW045	2023	2204
ZW046	2023	9105
ZW047	2023	53849
ZW048	2023	44119
ZW049	2023	118536
ZW050	2023	70049
ZW051	2023	7014
ZW052	2024	12200
ZW053	2024	28000
ZW054	2024	1914
ZW055	2024	32714
ZW056	2024	298646
ZW057	2024	61634
ZW058	2024	850426
ZW059	2024	144089
ZW060	2024	10385
ZW061	2024	97118
ZW062	2024	21405
ZW063	2024	118295
ZW064	2024	65458
ZW065	2024	11091
ZW066	2024	50320
ZW067	2024	6554
ZW068	2024	4136
ZW069	2024	153882
ZW070	2024	118295
ZW071	2024	164340
ZW072	2024	2541
ZW073	2024	17573
ZW074	2024	9724
ZW075	2024	26073
